# Superparamagnetic iron oxide nanoparticle restores gut microbiota homeostasis to enhance lung cancer immunotherapy

**DOI:** 10.1093/nsr/nwaf565

**Published:** 2025-12-15

**Authors:** Yayi He, Wengang Zhang, Zhanhang Guo, Wenbing Yu, Wencheng Zhao, Li Ye, Zhimin Chen, Yujie Li, Kandi Xu, Qianqian Zhang, Xinyue Liu, Yujin Liu, Hao Wang, Lishu Zhao, Xuyang Chen, Yuhang Li, Jingyi Sheng, Ning Gu

**Affiliations:** Institute of Clinical Medicine, Jiangsu Key Laboratory for Cardiovascular Information and Health Engineering Medicine, Nanjing Drum Tower Hospital, Medical School, Nanjing University, Nanjing 210093, China; Department of Medical Oncology, Shanghai Pulmonary Hospital, School of Medicine, Tongji University, Shanghai 200433, China; Department of Medical Oncology, Shanghai Pulmonary Hospital, School of Medicine, Tongji University, Shanghai 200433, China; Institute of Clinical Medicine, Jiangsu Key Laboratory for Cardiovascular Information and Health Engineering Medicine, Nanjing Drum Tower Hospital, Medical School, Nanjing University, Nanjing 210093, China; Yangtze River Delta Medical Advanced Technology Innovation Center, Taizhou 225403, China; Department of Medical Oncology, Shanghai Pulmonary Hospital, School of Medicine, Tongji University, Shanghai 200433, China; Department of Medical Oncology, Shanghai Pulmonary Hospital, School of Medicine, Tongji University, Shanghai 200433, China; Department of Medical Oncology, Shanghai Pulmonary Hospital, School of Medicine, Tongji University, Shanghai 200433, China; Department of Medical Oncology, Shanghai Pulmonary Hospital, School of Medicine, Tongji University, Shanghai 200433, China; Department of Medical Oncology, Shanghai Pulmonary Hospital, School of Medicine, Tongji University, Shanghai 200433, China; Department of Medical Oncology, Shanghai Pulmonary Hospital, School of Medicine, Tongji University, Shanghai 200433, China; Department of Medical Oncology, Shanghai Pulmonary Hospital, School of Medicine, Tongji University, Shanghai 200433, China; Department of Medical Oncology, Shanghai Pulmonary Hospital, School of Medicine, Tongji University, Shanghai 200433, China; Department of Medical Oncology, Shanghai Pulmonary Hospital, School of Medicine, Tongji University, Shanghai 200433, China; Department of Medical Oncology, Shanghai Pulmonary Hospital, School of Medicine, Tongji University, Shanghai 200433, China; Department of Medical Oncology, Shanghai Pulmonary Hospital, School of Medicine, Tongji University, Shanghai 200433, China; Department of Medical Oncology, Shanghai Pulmonary Hospital, School of Medicine, Tongji University, Shanghai 200433, China; Jiangsu Key Laboratory for Biomaterials and Devices, School of Biological Science and Medical Engineering, Southeast University, Nanjing 210009, China; Institute of Clinical Medicine, Jiangsu Key Laboratory for Cardiovascular Information and Health Engineering Medicine, Nanjing Drum Tower Hospital, Medical School, Nanjing University, Nanjing 210093, China

**Keywords:** superparamagnetic iron oxide nanoparticle, gut microbiota, lung cancer, immunotherapy

## Abstract

Emerging evidence indicates that gut microbiota dysbiosis markedly compromises the efficacy of lung cancer immunotherapy. In our study, superparamagnetic iron oxide nanoparticle assemblies (SPIOCAs) were developed and shown to effectively inhibit lung cancer growth at a dose of 12.5 mg/kg. Pretreatment with broad-spectrum antibiotics aggravates the gut dysbiosis that blunts programmed cell death protein 1 (PD-1) blockade in tumor-bearing mice, whereas SPIOCA administration reconstituted the gut microbiota and thereby resensitized tumors to anti-PD-1 therapy. SPIOCA gavage fortified intestinal barrier integrity—evidenced by elevated ZO-1, ZO-2, Occludin and Claudin-1 expression—and potentiated antitumor immune-cell infiltration, specifically by CD8+ T cells and dendritic cells, into the tumor microenvironment. We therefore preliminarily conclude that SPIOCAs restore gut microbiota homeostasis in lung cancer, thereby enhancing intestinal barrier integrity and converting the tumor immune microenvironment from an immune desert to an immune-inflamed phenotype, ultimately improving lung cancer immunotherapy efficacy.

## Teaser

Novel iron-oxide nanoparticles boost lung cancer immunotherapy by restoring gut health and awakening antitumor immunity, offering a promising approach to overcome immunotherapy resistance.

## INTRODUCTION

According to the 2022 GLOBOCAN data, lung cancer ranks as the most common malignant tumor globally in terms of both incidence and mortality rates [1]. Over the past decades, chemotherapy has been the standard first-line therapeutic option for advanced stages of both non-small cell lung cancer (NSCLC) and small cell lung cancer (SCLC) [2,3]. However, the 5-year survival rate for advanced lung cancer patients receiving standard chemotherapy is less than 5% [4].

The advent of immune checkpoint inhibitors (ICIs) that target programmed cell death protein 1 (PD-1) or its ligand (PD-L1) has dramatically transformed the therapeutic landscape for advanced lung cancer [5,6]. In the KEYNOTE-024 trial, the pembrolizumab group exhibited a substantially higher 5-year survival rate of 31%, compared to 16% in the chemotherapy group [7]. Results from a randomized controlled trial (RCT) of ASTRUM-005 indicated that first-line serplulimab (PD-1 inhibitor) plus platinum–etoposide [median overall survival (OS): 15.4 months] significantly prolonged OS compared to treatment with platinum–etoposide alone (median OS: 10.9 months) in extensive stage SCLC [8]. Other representative RCTs including IMpower133 [9], RATIONALE-312 [10], CASPIAN [11], CAPSTONE-1 [12], KEYNOTE-407 [13], RATIONALE-307 [14], CheckMate-9LA [15] and GEMSTONE-302 [16], among others, have yielded similar findings. Nevertheless, the response rate to immunotherapy remains limited, with only approximately 20%–30% of patients achieving a favorable response. The underlying mechanisms of immunotherapy resistance remain obscure. Therefore, there is a great urgency to elucidate the mechanisms of immunotherapy resistance and explore effective strategies to improve immunotherapy efficacy.

Numerous studies have been devoted to exploring the mechanisms that modulate the efficacy of immunotherapy. Recent research has identified that PD-L1 expression levels, tumor immunity within the microenvironment, antigen loss through immunoediting, defects in tumor antigen presentation, and metabolic alterations are all closely correlated with the efficacy of immunotherapy in lung cancer [17,18]. Interestingly, a growing body of research has underscored the vital role of the gut microbiota in modulating the effectiveness of immunotherapy [19]. The human gut microbiota is a complex ecosystem of bacteria, fungi, viruses, archaea and parasites, with microbial cells usually outnumbering host cells [20]. The gut microbiota harbors a gene repertoire that is over 100 times larger than the human genome, thereby representing a far greater degree of genetic diversity and earning its designation as the ‘second human genome’ [21]. The gut microbiota’s role in the regulation of innate immunity, as well as adaptive immunity, has been well documented [22]. Accumulating evidence underscores the crucial influence of gut microbiota homeostasis in tumor development, progression and therapeutic responses [20]. A prime example is the mounting evidence from both preclinical and clinical studies that shows how the gut microbiota can affect responses to ICIs [23–25]. For example, in lung cancer patients treated with anti-PD-1 therapy, a notable variation in the diversity and composition of the gut microbiota was found between those who responded well and those who did not [26]. Germ-free mice that received fecal microbiota transplantation (FMT) from responders displayed enhanced responsiveness to anti-PD-1 treatment relative to those that received FMT from non-responders [27,28]. Moreover, clinical trials have confirmed that FMT can boost the effectiveness of immunotherapy. Davar *et al*. revealed that FMT has the potential to modify the gut microbiota and reshape the tumor microenvironment (TME), thereby overcoming resistance to anti-PD-1 therapy in patients with advanced melanoma [29,30]. Notwithstanding this, the clinical application of FMT is hindered by challenges including standardization of protocols, donor screening, safety concerns and long-term efficacy [31]. Therefore, there is a pressing clinical need for alternative strategies to safely and effectively restore gut microbiota homeostasis.

Mounting evidence indicates that the structure, function and diversity of the gut microbiota are profoundly impacted by diet [32–34]. The fasting-mimicking diet was demonstrated to enrich *Bifidobacterium pseudolongum*, thereby promoting the infiltration of CD8+ T cells and exerting a suppressive effect on colorectal cancer [35]. Studies have demonstrated that iron is essential for regulating and maintaining gut microbiota homeostasis [36]. In our prior work, we developed a pH-responsive superparamagnetic iron oxide nanoparticle assembly (SPIOCA) coated with carboxymethyl cellulose (CMC), an excipient approved by the Food and Drug Administration (FDA), and revealed that SPIOCA supplementation could effectively reshape gut microbiota dysbiosis and hold promise for treating noise-induced hearing loss [37]. At present, the interplay between iron, the gut microbiota and tumor immunotherapy remains inadequately explored. Building on our prior discovery that a SPIOCA can effectively modulate the gut microbiota, we propose that SPIOCAs may hold the potential for enhancing lung cancer immunotherapy via gut microbiota regulation.

In this study, we first compared the gut microbiota structure between healthy individuals and lung cancer patients and found that lung cancer patients exhibited gut microbiota dysbiosis with reduced diversity. Based on our previously developed pH-responsive SPIOCA [37], we then explored its effects on maintaining gut microbiota homeostasis and enhancing lung cancer immunotherapy efficacy through *in vivo* experiments. Furthermore, the mechanisms underlying its impact on lung cancer immunotherapy were elucidated by evaluating its regulatory effects on gut barrier function and the TME.

## RESULTS

### The diversity and composition of the gut microbiota between lung cancer and healthy individuals

**Figure 1.**
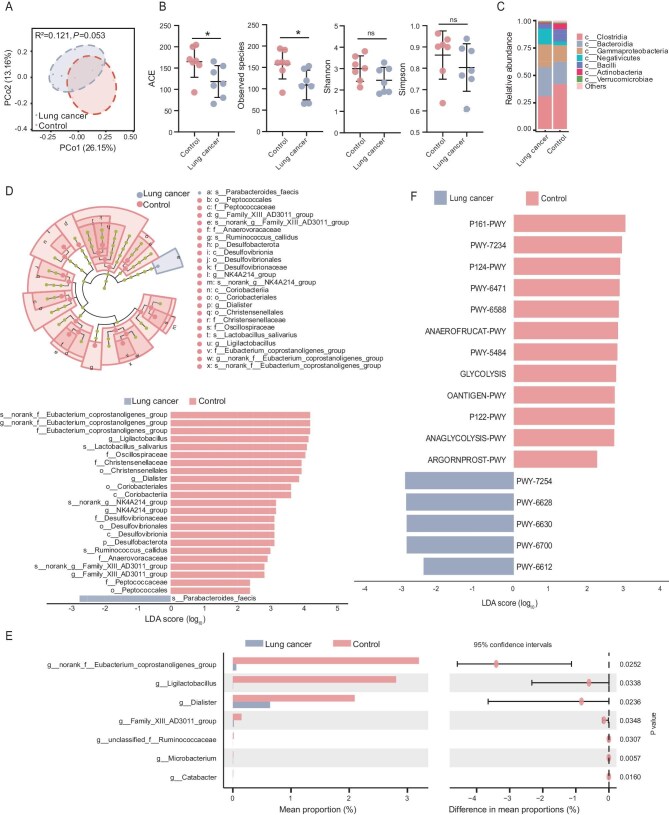
The diversity and composition of the gut microbiota between the lung cancer and healthy individuals. (A) Microbial β-diversity PCoA analysis between the lung cancer patients (*n* = 7) and healthy individuals (*n* = 7). (B) Comparison of bacterial community α-diversity indices (including ACE, observed species, Shannon and Simpson) between lung cancer patients and healthy individuals was conducted using the Wilcoxon test. (C) Bar plot illustrating the relative abundance of gut microbiota at the class level. (D) Cladogram generated through LEfSe with corresponding LDA scores, depicting the distinct gut microbiota profiles in lung cancer patients versus healthy individuals. (E) Comparison of relative taxon abundance at the genus level between lung cancer and control groups based on the Wilcoxon rank-sum test. (F) MetaCyc pathway enrichment analysis in lung cancer versus control groups. ns, not significant; * *P* < 0.05.

Fecal samples from seven newly diagnosed lung cancer patients and seven healthy controls were collected for 16S rRNA sequencing, with demographic and clinical information provided in Table S1. The plateauing of the Shannon–Wiener index curve (Fig. S1) suggests that the sequencing depth is adequate to reflect the true microbial diversity. Principal coordinates analysis (PCoA) indicated that the bacterial community compositions of lung cancer patients and healthy individuals tend to diverge (Fig. 1A). The α-diversity of the bacterial community was higher in healthy individuals, with abundance-based coverage estimator (ACE) and observed species significantly elevated compared to that in those with lung cancer, and Shannon and Simpson indices trended higher but without statistical significance (Fig. 1B). Figure 1C shows that, at the class level, the gut microbiota of healthy individuals is enriched in Clostridia, Bacilli and Actinobacteria, while that of lung cancer patients is characterized by higher abundances of Bacteroidia, Gammaproteobacteria and Negativicutes. Linear discriminant analysis effect size (LEfSe) revealed that potential probiotics associated with short-chain fatty acid (SCFA) metabolism and immune regulation, including *Eubacterium* species, *Ruminococcus callidus, Ligilactobacillus* species, *Lactobacillus salivarius*, Oscillospiraceae and Christensenellaceae, were significantly reduced in lung cancer patients compared to healthy controls (Fig. 1D). Wilcoxon rank-sum test analysis at the genus level revealed comparable microbial disparities, with *Eubacterium* and *Ligilactobacillus* species exhibiting significantly higher abundance in healthy individuals than in lung cancer patients (all *P* < 0.05) (Fig. 1E). Consistently, LEfSe analysis of metabolic pathways revealed that several pathways involved in maintaining gut microbiota homeostasis, particularly those associated with energy metabolism and SCFA production, including ANAGLYCOLYSIS-PWY, P122-PWY, ANAEROFRUCAT-PWY and PWY-6588, were significantly enriched in healthy individuals compared to lung cancer patients (Fig. 1F).

### Inhibition of lung cancer and gut microbiota remodeling by a SPIOCA

**Figure 2.**
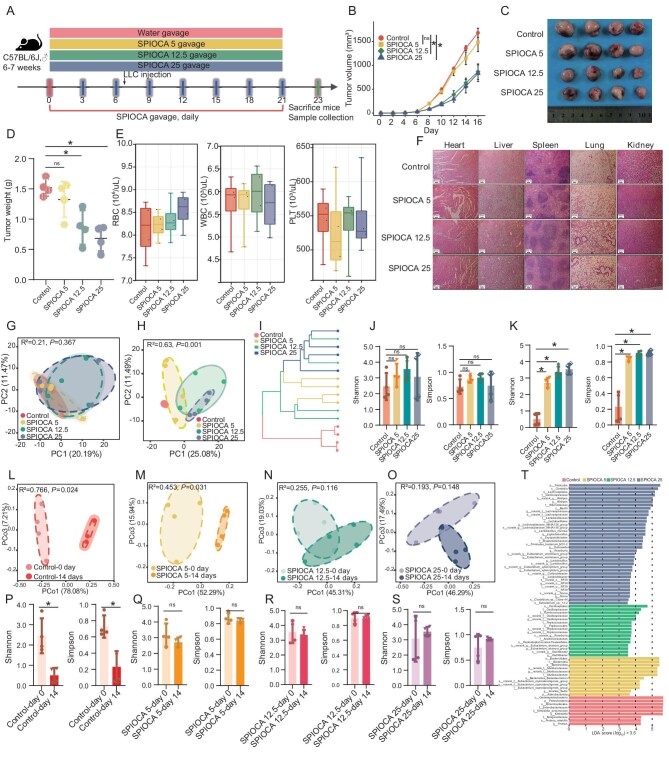
Inhibition of lung cancer and gut microbiota remodeling by the SPIOCA. (A) The experimental design for administering different doses of SPIOCA by gavage in the lung cancer-bearing mouse model. (B) Growth curves of subcutaneous tumors were analyzed across different SPIOCA dosing groups (*n* = 4) using two-way ANOVA (SPIOCA at 0, 5, 12.5 and 25 mg/kg iron doses). (C) Images of tumors excised from mice on Day 23 after sacrifice. (D) Comparison of tumor weights among different groups following euthanasia on Day 23 was performed using one-way ANOVA. (E) Bar plots illustrating the levels of red blood cells (RBCs), white blood cells (WBCs) and platelets (PLTs) in peripheral blood across distinct groups of mice. (F) Images of the H&E-stained sections of main organs. (G and H) Bacterial community compositions among groups before (G) and after (H) SPIOCA treatment, as assessed by PCA. (I) Cluster dendrogram in four groups. (J and K) Comparative analysis of Shannon and Simpson indices of bacterial communities in four groups before (J) and after (K) SPIOCA gavage using the Wilcoxon test. (L–O) Comparison of bacterial community compositions before and after SPIOCA treatment within each group [control (L), SPIOCA 5 (M), SPIOCA 12.5 (N) and SPIOCA 25 (O)] based on PCoA analysis. (P–S) Comparison of the α-diversity indices (Shannon and Simpson) before and after SPIOCA treatment within each group [control (P), SPIOCA 5 (Q), SPIOCA 12.5 (R) and SPIOCA 25 (S)] using the Wilcoxon test. (T) Differential abundance of microbiota across the four groups, as determined by LEfSe analysis (LDA score > 2 and *P* < 0.05). ns, not significant; * *P* < 0.05.

To evaluate the antitumor efficacy and biosafety of a SPIOCA, we administered different doses of the SPIOCA (0, 5, 12.5 and 25 mg/kg iron dose) via daily oral gavage to lung cancer-bearing C57BL/6J mice (Fig. 2A). The SPIOCA demonstrated significant anti-tumor activity against lung cancer growth at doses of 12.5 and 25 mg/kg, which was comparable between these two doses and significantly greater than that at 5 mg/kg (*P* < 0.05) and in the control group (*P* < 0.05) (Fig. 2B and C). Consistent with tumor volume, tumor weight was significantly reduced in the SPIOCA 12.5 and 25 mg/kg groups compared to the 5 mg/kg and control groups (*P* < 0.05) (Fig. 2D). These findings were recapitulated when the cohort size was increased to seven mice per group (Fig. S2). Peripheral blood analyses (Fig. 2E) and histological assessments (Fig. 2F) of major organs (heart, liver, spleen, lung and kidney) using hematoxylin and eosin (H&E) staining across all groups revealed no significant adverse effects or pathological changes, confirming the biosafety of the SPIOCA.

Fecal samples from mice were collected before and after SPIOCA gavage for 16S rRNA sequencing. Principal component analysis (PCA) showed no significant differences in bacterial community compositions among groups prior to SPIOCA treatment (Fig. 2G). In contrast, distinct differences in gut microbiota composition were observed among the four groups following SPIOCA gavage (*P* = 0.001) (Fig. 2H). Hierarchical clustering analysis further revealed clear clustering patterns, with SPIOCA-treated samples forming a cohesive cluster and control samples clustering separately (Fig. 2I), indicating significant alterations in microbial profiles induced by SPIOCA treatment. The α-diversity indices (Shannon and Simpson) of bacterial communities were similar among groups before SPIOCA gavage (Fig. 2J), but significantly lower in the control group compared to SPIOCA-treated groups after treatment (*P* < 0.05) (Fig. 2K). Further comparison of gut microbiota structure before and after SPIOCA gavage revealed distinct differences in the control and SPIOCA 5 mg/kg groups (Fig. 2L and M), whereas no significant differences were observed in the SPIOCA 12.5 mg/kg and 25 mg/kg groups (Fig. 2N and O). Regarding α-diversity indices, in the control group, both Shannon and Simpson indices significantly decreased after tumor implantation compared to pre-tumor levels (Fig. 2P). In contrast, in SPIOCA-treated groups, no significant differences in Shannon and Simpson indices were observed between pre- and post-tumor implantation stages, regardless of the dose (Fig. 2Q–S). LEfSe analysis demonstrated that probiotics associated with gut microbiota metabolism and immune regulation, such as Firmicutes, Lachnospirales, Rikenellaceae, Lactobacillaceae, *Lactobacillus* species, *Ligilactobacillus* species, Erysipelotrichaceae, *Roseburia murinus, Eubacterium xylanophilum, Dorea* species, *Clostridium* species and Oscillospirales, were significantly enriched in the SPIOCA group (Fig. 2T). In contrast, potentially pathogenic taxa, including Gammaproteobacteria, Proteobacteria, Enterobacteriaceae, *Klebsiella* species and *Proteus mirabilis*, were more prevalent in the control group (Fig. 2T).

### SPIOCA synergizes with lung cancer immunotherapy via gut microbiota and metabolite remodeling

**Figure 3.**
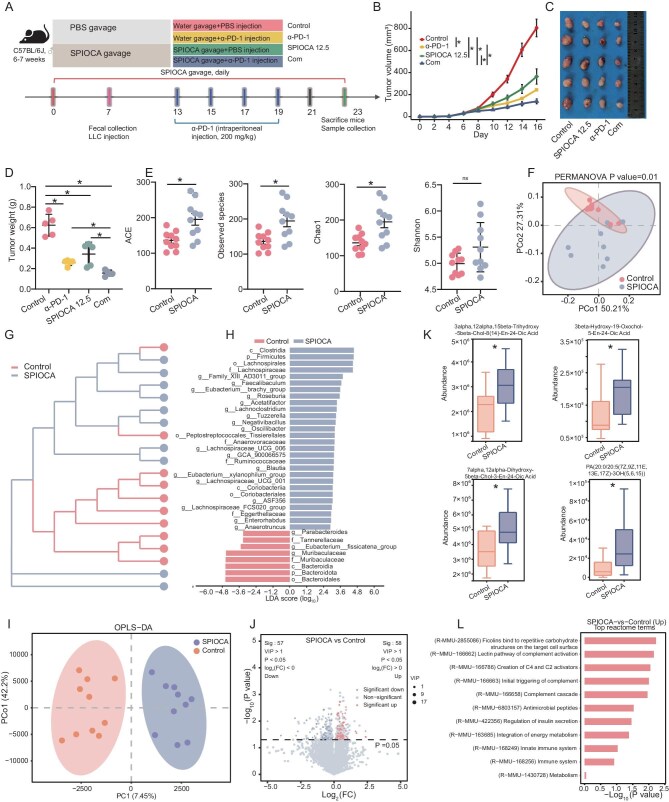
The synergistic effects of SPIOCA and PD-1 inhibitors, as well as their impacts on the structure and metabolism of gut microbiota. (A) Schematic diagram of the mouse experimental process involving treatment with SPIOCA and PD-1 inhibitors. (B) Growth curves of subcutaneous tumors were analyzed across different groups: control, α-PD-1, SPIOCA 12.5 and combination (Com), with five mice per group, using two-way ANOVA. (C) Images of tumors excised from mice on Day 23 after sacrifice. (D) Comparison of tumor weights among control, α-PD-1, SPIOCA 12.5 and Com groups after sacrifice on Day 23 using one-way ANOVA. (E) The comparison of α-diversity indices, including ACE, observed species, Chao1 and Shannon, across four distinct groups using the Wilcoxon test. (F and G) The cluster dendrogram and PCoA analyses of microbial profiles between the SPIOCA and control groups. (H) Differential abundance of microbiota between the SPIOCA and control groups, as determined by LEfSe analysis (|LDA score| > 2 and *P* < 0.05). (I) Comparison of metabolic profiles between the SPIOCA andcontrol groups using orthogonal partial least squares discriminant analysis (OPLS-DA). (J) Volcano plot showing metabolites with differential abundance in the SPIOCA and control groups. (K) Bar graph depicting the metabolites that are significantly upregulated in the SPIOCA group relative to the control group. (L) Reactome enrichment analysis in the SPIOCA group versus the control group. ns, not significant; * *P* < 0.05; ** *P* < 0.01.

The potential synergy between the SPIOCA (12.5 mg/kg) and PD-1 blockade was assessed (Fig. 3A). Both SPIOCA and PD-1 inhibitor monotherapy effectively slowed tumor growth relative to controls, with the most significant reduction observed in the combined treatment group (Fig. 3B and C). These findings were corroborated by tumor weight measurements (Fig. 3D). The α-diversity indices, including ACE, observed species and Chao1, were significantly higher in the SPIOCA group compared to the control group, while the Shannon index showed a tendency towards higher values in the SPIOCA group, albeit without statistical significance (Fig. 3E). The cluster dendrogram and PCoA revealed distinct microbial profiles between the SPIOCA and control groups (Fig. 3F and G). LEfSe analysis indicated that SPIOCA administration markedly increased the abundance of key probiotics linked to SCFA production, gut barrier integrity and immune regulation, such as Clostridia, Lachnospirales, *Roseburia* species, *Acetatifactor* species, *Lachnoclostridium* species, *Faecalibaculum* species and *Eubacterium brachy* (Fig. 3H). Further non-targeted metabolomic analysis of fecal samples demonstrated distinct metabolic profiles between the SPIOCA and control groups (Fig. 3I), with 58 metabolites significantly upregulated and 57 metabolites downregulated in the SPIOCA group (Fig. 3J). In the SPIOCA-treated group, metabolites involved in maintaining intestinal barrier function and immune regulation, including 7α,12α-dihydroxy-5β-chol-3-en-24-oic acid, 3α,12α,15β-trihydroxy-5β-chol-8(14)-en-24-oic acid, 3β-hydroxy-19-oxochol-5-en-24-oic acid and PA(20:0/20:5(7Z,9Z,11E,13E,17Z)-3OH(5,6,15)), were significantly upregulated (Fig. 3K). In the Reactome enrichment analysis, pathways related to complement activation, immune regulation and energy metabolism, including ‘Ficolins bind to repetitive carbohydrate structures’, ‘Lectin pathway of complement activation’, ‘Creation of C4 and C2 activators’, ‘Integration of energy metabolism’ and ‘Innate immune system’ were significantly enriched in the SPIOCA group (Fig. 3L).

**Figure 4.**
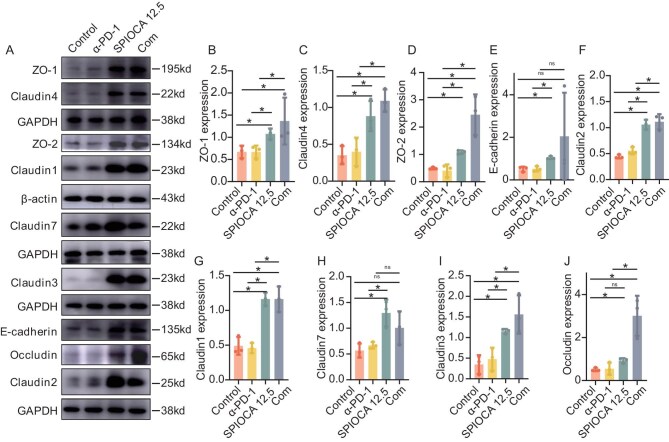
Evaluation of the impact of the SPIOCA on intestinal barrier function. (A) Western blotting detection of gut barrier-associated proteins in intestinal tissues from control, α-PD-1, SPIOCA 12.5 and Com groups. (B–J) Bar plots showing the grayscale value statistics of Western blot results for various gut barrier-associated proteins in (A) using one-way ANOVA. ns, not significant; * *P* < 0.05.

Given the enrichment of gut barrier-associated microbiota in the SPIOCA group, we assessed intestinal barrier function across groups by examining corresponding markers. Western blotting analysis revealed that the expression levels of Claudin-2, Claudin-3, Claudin-7, Claudin-1, Claudin-4, E-cadherin, Occludin, ZO-1 and ZO-2 were higher in intestinal tissues from the SPIOCA group (including both monotherapy and combination with PD-1 inhibitors) than in those from the control or PD-1 inhibitor group (Fig. 4A–J).

### SPIOCA restores lung cancer immunotherapy response and reverses gut dysbiosis in the Antibiotic cocktail (Abx) model

**Figure 5.**
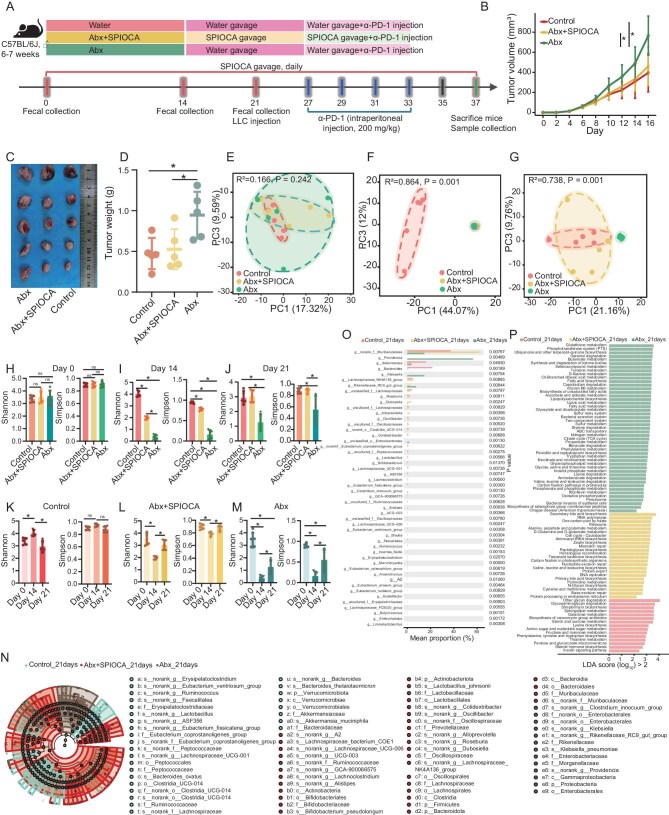
The SPIOCA restores lung cancer immunotherapy response and reverses gut dysbiosis in the Abx model. (A) Schematic diagram of the construction of the Abx model and intervention. (B) Growth curves of subcutaneous tumors were evaluated in different groups: control, Abx + SPIOCA and Abx, with five mice per group, using two-way ANOVA to determine statistical differences between the groups. (C) Images of tumors excised from mice on Day 37 after sacrifice. (D) Comparison of tumor weights among control, Abx + SPIOCA and Abx groups after sacrifice on Day 37 using one-way ANOVA. (E–G) Comparison of bacterial community compositions among control, Abx + SPIOCA and Abx groups using fecal samples collected on Day 0 (E), Day 14 (F) and Day 21 (G), based on PCA. (H–J) Comparison of α-diversity indices (Shannon and Simpson) among control, Abx + SPIOCA and Abx groups using fecal samples collected on Day 0 (H), Day 14 (I) and Day 21 (J), based on the Wilcoxon test. (K–M) Comparison of the α-diversity indices (Shannon and Simpson) based on fecal samples collected from the same group [control (K), Abx + SPIOCA (L), Abx (M)] of mice at different timepoints (Day 0 vs. Day 14 vs. Day 21) using the Wilcoxon test. (N) LEfSe taxonomic cladogram showing associations among microbiota communities in the control, Abx + SPIOCA and Abx groups. Each node represents a specific taxonomic type. Yellow nodes denote the taxonomic features that are not significantly differentiated across groups. Red nodes denote taxonomic types with higher abundance in the Abx + SPIOCA group. Green nodes represent those more abundant in the control group. Brown nodes indicate taxonomic types enriched in the Abx group. (O) Comparison of relative taxon abundance at the genus level among control, Abx + SPIOCA and Abx groups using fecal samples collected on Day 21, based on the Kruskal–Wallis test. (P) KEGG enrichment analysis among control, Abx + SPIOCA and Abx groups using fecal samples collected on Day 21. * *P* < 0.05.

To further investigate the regulatory and stabilizing effects of the SPIOCA on the gut microbiota, we established the dysbiosis model by administering an Abx (Fig. 5A). Compared with the control group, the Abx group exhibited significantly accelerated tumor growth, yet the addition of the SPIOCA effectively restored the antitumor efficacy of PD-1 inhibitors that had been diminished by Abx treatment (Fig. 5B and C). Tumor weight exhibited a comparable outcome (Fig. 5D).

Before Abx administration, the gut microbiota profiles were comparable among the three groups (Fig. 5E). Following 2 weeks of Abx treatment, the microbiota in the Abx and Abx + SPIOCA groups converged, showing clear divergence from the control group (Fig. 5F). One week after discontinuation of Abx treatment, the gut microbial community in the Abx + SPIOCA group normalized to resemble the control group, differing significantly from the Abx group (Fig. 5G). Before Abx treatment, α-diversity indices were comparable across all groups (Fig. 5H). At 2 weeks post-treatment, α-diversity was lowest in the Abx group, intermediate in the Abx + SPIOCA group, and highest in controls (Fig. 5I). One week after Abx cessation, the Abx + SPIOCA group recovered to levels comparable to the control group, both of which were significantly higher than the Abx group (Fig. 5J). The α-diversity indices of each group were compared at different timepoints. For the control group, the Shannon index was significantly higher at Day 14 than at baseline but decreased at Day 21, while the Simpson index showed no significant differences across timepoints (Fig. 5K). For the Abx + SPIOCA group, α-diversity indices significantly decreased after Abx administration but recovered to baseline levels 7 days after Abx cessation (Fig. 5L). For the Abx group, indices also decreased significantly after Abx treatment, partially recovered 7 days post-cessation, but remained significantly lower than at baseline (Fig. 5M). The taxonomic cladogram derived from LEfSe analysis indicated that several probiotic taxa, including *Roseburia, Colidextribacter, Oscillibacter*, Lachnospiraceae NK4A136 group, *Eubacterium ventriosum* group and *Akkermansia muciniphila*, were significantly more abundant in the control and SPIOCA + Abx groups compared to the Abx group (Fig. 5N and O). KEGG enrichment analysis highlighted significant enrichment of various metabolism-related pathways, including primary and secondary bile acid biosynthesis, one-carbon metabolism via folate, biosynthesis of branched-chain amino acids (valine, leucine and isoleucine), in the control and SPIOCA + Abx groups (Fig. 5P).

### SPIOCA restores gut barrier function and remodels the lung cancer immune microenvironment

**Figure 6.**
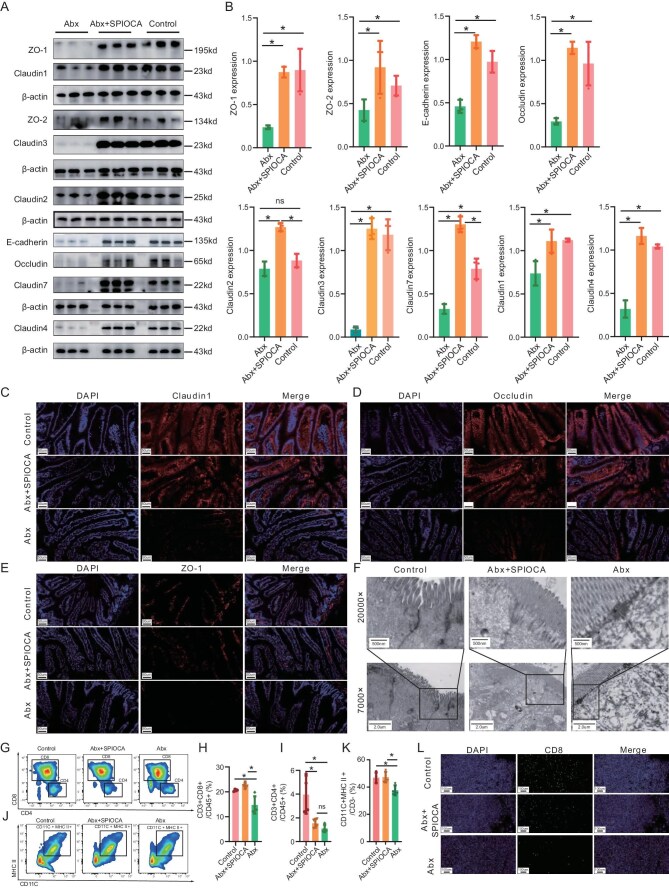
Assessment of the SPIOCA’s impact on gut barrier function and the lung-cancer immune microenvironment. (A) Western blotting detection of gut barrier-associated proteins in intestinal tissues from control, Abx + SPIOCA and Abx groups. (B) Bar plots illustrate the grayscale value statistics of Western blot results for various gut barrier-associated proteins (A), analyzed using one-way ANOVA. (C–E) Immunofluorescence analysis for the representative tight junction proteins claudin-1 (C), occludin (D) and ZO-1 (E) using intestinal tissues from control, Abx + SPIOCA and Abx groups. Scale bar = 50 µm. (F) Representative images of intercellular junctions in intestinal tissues isolated from Abx + SPIOCA and Abx mice, visualized by TEM. (G) Flow cytometric analysis of CD4+ T cells and CD8+ T cells utilizing tumor tissues isolated from control, Abx + SPIOCA and Abx mice. (H) Quantitative analysis of CD3+CD8+ T cells using one-way ANOVA. (I) Quantitative analysis of CD3+CD4+ T cells using one-way ANOVA. (J) Flow cytometric analysis of DCs utilizing tumor tissues isolated from control, Abx + SPIOCA and Abx mice. (K) Quantitative analysis of CD11C+MHC II+ DCs using one-way ANOVA. (L) Micrographs of immunofluorescence staining of immune markers [4′,6-diamidino-2-phenylindole (DAPI), CD8] in tumor tissues isolated from control, Abx + SPIOCA and Abx mice. ns, not significant; * *P* < 0.05.

To evaluate the effects of the SPIOCA on intestinal barrier function, we assessed the expression of intestinal barrier-related markers, including Claudin-2, Claudin-3, Claudin-7, Claudin-1, Claudin-4, E-cadherin, Occludin, ZO-1 and ZO-2, using Western blotting analysis. Results showed that the expression levels of these markers were comparable between the Abx + SPIOCA and control groups and were significantly higher than those in the Abx group (Fig. 6A and B). Subsequent immunofluorescence staining corroborated these findings, with Claudin-1 (Fig. 6C), Occludin (Fig. 6D) and ZO-1 (Fig. 6E) expression higher in the Abx + SPIOCA and control groups than in the Abx group, further confirming that SPIOCA treatment effectively preserves or restores intestinal barrier integrity. To further assess the effects of the SPIOCA on cell–cell junction integrity, transmission electron microscopy (TEM) was employed to visualize the intestinal cellular junctions in different groups. As expected, a significantly larger gap between intestinal epithelial cells was observed in the Abx group (Fig. 6F, right panel), whereas the integrity of the intestinal epithelial cell junction was preserved in both the control (Fig. 6F, left panel) and Abx + SPIOCA groups (Fig. 6F, middle panel).

Based on our previous findings that the SPIOCA modulates the gut microbiota and metabolism, we speculate that it may further reshape the TME to enhance the response to PD-1 inhibitors. Initially, immune cell (CD4+ T cell and CD8+ T cell) infiltration in tumors was assessed across different groups by flow cytometry. The percentage of CD3+CD8+/CD45+ cells was comparable between the control and SPIOCA + Abx groups, yet significantly higher than that in the Abx group (Fig. 6G and H). Compared with both the α-PD-1 and control groups, the combination and SPIOCA cohorts displayed a markedly higher proportion of CD8+GZMB+ T cells (Fig. S3). However, the percentage of CD3+CD4+/CD45+ cells was comparable between the SPIOCA + Abx and Abx groups, both of which were significantly lower than that in the control group (Fig. 6G and I). Given the critical role of dendritic cells (DCs) in immunotherapy responses, the infiltration of CD11c+MHC II+/CD3− cells was also evaluated (Fig. 6J), revealing significantly higher levels in the control and SPIOCA + Abx groups compared to the Abx group (Fig. 6K). The above hypothesis was further corroborated by immunofluorescence staining, which revealed higher positivity for CD8 in the control and SPIOCA + Abx groups compared to the Abx group (Fig. 6L).

## DISCUSSION

Given the low response rates to immunotherapy in lung cancer, extensive research has been dedicated to elucidating the mechanisms that regulate its efficacy and exploring strategies to enhance immunotherapy [38]. Currently, multiple factors have been identified that modulate the efficacy of immunotherapy in lung cancer, among which the gut microbiota has increasingly been recognized as a crucial determinant [26]. Based on our previous findings that SPIOCAs regulate and maintain gut microbiota homeostasis [37], we hypothesized that a SPIOCA could modulate the response to lung cancer immunotherapy by influencing the gut microbiota. In this study, we comprehensively demonstrated the synergistic antitumor effects and tolerable biosafety of a SPIOCA in combination with PD-1 inhibitors in lung cancer through *in vivo* experiments. Fecal 16S rRNA sequencing and non-targeted metabolomics at different timepoints supported that SPIOCA effectively maintains and restores gut microbiota homeostasis and modulates microbial metabolism. These findings suggest that a SPIOCA is a safe and effective strategy with the potential to enhance lung cancer immunotherapy in future clinical applications.

An increasing number of studies have shown that the gut microbiota is closely associated with cancer initiation, progression and metastasis [20,39]. In lung cancer patients, the gut microbiota exhibits characteristic alterations compared to healthy individuals [40]. Specifically, the abundance of Firmicutes is reduced, while the abundance of Proteobacteria and Bacteroidetes is increased [41,42]. In this study, consistent with previous findings, 16S rRNA sequencing revealed that lung cancer patients exhibited reduced α-diversity compared to healthy individuals. Moreover, the abundance of potentially pathogenic bacteria, such as Bacteroidia, Gammaproteobacteria and Negativicutes, was higher in lung cancer patients. In contrast, healthy individuals had higher levels of probiotics associated with immune regulation and SCFA metabolism, including *Eubacterium* species, *Ruminococcus callidus, Ligilactobacillus* species, *Lactobacillus salivarius*, Oscillospiraceae and Christensenellaceae. Consistent with these findings, our mouse experiments revealed that tumor-bearing mice exhibited reduced gut microbiota diversity, accompanied by a decrease in the abundance of potential probiotics. These findings further highlight the distinct gut microbiota patterns in lung cancer patients and suggest that modulating the gut microbiota could be a potential strategy for improving treatment outcomes.

Past research has extensively explored the modulation of tumor therapy through the gut microbiota in both basic and clinical studies [19,43]. In a mouse experiment, Routy *et al*. demonstrated that FMT from cancer patients who responded to immunotherapy enhanced the antitumor effects of PD-1 blockade in germ-free or antibiotic-treated mice [44]. Davar *et al*. conducted a clinical trial that demonstrated FMT could overcome resistance to anti-PD-1 therapy in melanoma patients [29]. Nevertheless, the implementation of FMT in clinical settings is impeded by several factors, including the transmission of pathogens, immune rejection and the scarcity of donors [31]. Iron, an essential metal element vital for human health, is indispensable and needs to be supplemented through external sources. Emerging evidence has demonstrated that iron can profoundly reshape the gut microbiota and modulate host metabolism [45]. In our preliminary work, we developed a pH-responsive SPIOCA coated with FDA-approved CMC, which ensures superior stability and demonstrates the ability to modulate gut microbiota homeostasis [37]. In this study, we propose that a SPIOCA may enhance the efficacy of PD-1 inhibitors in lung cancer. In our *in vivo* experiments, we observed significant tumor growth inhibition at a dosage of 12.5 mg/kg. Consequently, a dosage of 12.5 mg/kg was administered in all subsequent animal trials. Subsequent studies further demonstrated that the SPIOCA synergistically enhanced the efficacy of PD-1 inhibitors and restored the diminished therapeutic efficacy of PD-1 inhibitors in Abx-treated mice, which was attributed to the restoration of gut microbiota homeostasis.

Utilizing 16S rRNA gene sequencing across multiple timepoints, we demonstrated that the SPIOCA has the capacity to preserve and re-establish gut microbiota equilibrium. Upon tumor inoculation, mice exhibited a significant reduction in gut microbiota α-diversity, yet SPIOCA administration effectively mitigated this decline and preserved homeostasis. These findings were further corroborated in Abx-treated mice, where the α-diversity of gut microbiota was significantly reduced compared to controls. However, in mice treated with Abx and SPIOCA, the decline in α-diversity was more moderate, and a rapid recovery was observed following cessation of Abx treatment. Microbial profiling revealed that several potential probiotics were significantly enriched following SPIOCA administration. For instance, in the SPIOCA-treated group, *Roseburia* species, anaerobic bacteria belonging to the phylum Firmicutes and known for their ability to produce SCFAs [46], particularly acetate, propionate and butyrate, was significantly enriched. *Faecalibaculum* species, within the phylum Firmicutes [47], were also enriched in the SPIOCA-treated group, producing SCFAs such as acetate, propionate and butyrate, which are essential for maintaining gut barrier function, modulating immune responses and providing energy to the host. Bile acids, common and crucial metabolites in the gut, have been shown to activate the G protein-coupled receptor TGR5, which promotes the repair and maintenance of the gut barrier. Additionally, bile acids can modulate the composition of the gut microbiota, thereby maintaining the integrity of the gut barrier. In our study, the levels of bile acid-related metabolites, including 7α,12α-dihydroxy-5β-chol-3-en-24-oic acid, 3α,12α,15β-trihydroxy-5β-chol-8(14)-en-24-oic acid, and 3β-hydroxy-19-oxochol-5-en-24-oic acid, were significantly higher in the SPIOCA group than in the control group, indicating that the SPIOCA may protect the intestinal barrier by modulating bile acid metabolism. The proteins Claudin-2, Claudin-3, Claudin-7, Claudin-1, Claudin-4, E-cadherin, Occludin, ZO-1 and ZO-2 are key markers associated with intestinal barrier function [48]. Our results revealed that the expression levels of these proteins were significantly elevated in the SPIOCA group compared to the control group. This finding supports our hypothesis that the SPIOCA protects intestinal barrier integrity by modulating the gut microbiota and metabolites critical for maintaining intestinal barrier function. The infiltration of immune cells in the TME is a critical factor limiting the efficacy of immunotherapy [49]. Therefore, the infiltration of key immune cells such as T lymphocytes and DCs was evaluated. Our results showed that CD8+ T cells, CD4+ T cells and DCs were significantly infiltrated in the SPIOCA group. Based on these findings, we conclude that the SPIOCA maintains intestinal barrier function by modulating the gut microbiota and metabolites, thereby promoting the infiltration of antitumor immune cells and synergizing with immunotherapy.

Although our study has developed and comprehensively demonstrated the ability of a SPIOCA to synergize with PD-1 inhibitors to enhance anti-tumor effects in lung cancer, there are still limitations that require further in-depth exploration in future research. Firstly, our study hypothesizes that the SPIOCA reshapes the TME and impacts immunotherapy by modulating gut microbiota metabolites, yet validation of specific metabolites remains to be conducted. Secondly, the differential impact of the SPIOCA versus iron alone remains unexplored and awaits future investigation. Thirdly, the lung-cancer cohort is too small and demands validation in a larger sample. Additionally, its safety and efficacy in humans have yet to be validated in prospective clinical trials. We are applying to undertake clinical trials to explore its safety and efficacy, and will collect patient samples to delve into its impact on microbiota structure, metabolism and the TME, thereby elucidating its mechanisms and facilitating clinical translation.

## CONCLUSION

The SPIOCA regulated the gut microbiota and metabolites, enhanced gut barrier function and shifted the TME from an immune desert to an immune-inflamed phenotype with increased anti-tumor immune cell infiltration, thereby improving the efficacy of lung cancer immunotherapy.

## METHODS

Statistical analyses and graphical representations were conducted using GraphPad Prism 9.0 (GraphPad Software Inc., La Jolla, CA, USA) and R (version 4.1.3). Western blot images were processed using ImageJ 1.8.0, and flow cytometry data were analyzed with FlowJo 10.8.1. Group comparisons were performed using analysis of variance (ANOVA) followed by two-group *t*-tests or Wilcoxon rank sum tests. Multiple group comparisons were assessed via one-way ANOVA, two-way ANOVA or Kruskal–Wallis tests with *post hoc* analyses. Bonferroni’s correction was applied to control type I errors in *post hoc* multiple comparisons. A *P* value of less than 0.05 was considered statistically significant, and all *P* values were two-tailed. The detailed methods and materials are available in the Supplementary Methods.

## Ethical statements

This work was performed in accordance with the recommendations in the *Guide for the Care and Use of Laboratory Animals* and relevant Chinese laws and regulations. All the animal assays were approved by the Institutional Animal Care and Use Committees of Tongji University (approval number: TJBE00524101).

## Supplementary Material

nwaf565_Supplemental_File
