# Supplementary material for: Superparamagnetic iron oxide nanoparticle restores gut microbiota homeostasis to enhance lung cancer immunotherapy
Source: Natl Sci Rev. 2025 Dec 15;13(3):nwaf565. doi: 10.1093/nsr/nwaf565 (PMC12875120; doi:10.1093/nsr/nwaf565)
Supplement: nwaf565_Supplemental_File [file nwaf565_supplemental_file.pdf]

Supplementary Data 1

Supplementary Table 1. Clinical characteristics of the 7 lung cancer cases and 7 controls.

Variables	Control (n = 7)	Lung cancer (n = 7)
Age, Mean ± SD	64.71 ± 6.24	65.29 ± 7.87
Sex, n(%)		
Female	1 (14.29)	1 (14.29)
Male	6 (85.71)	6 (85.71)
Histology, n(%)		
LUAD	/	4 (57.14)
LUSC	/	1 (14.29)
NSCLC	/	2 (28.57)
Stage, n(%)		
IVB	/	7 (100.00)

LUAD: lung adenocarcinoma; LUSC: lung squamous cell carcinoma; NSCLC: non-small cell lung cancer.

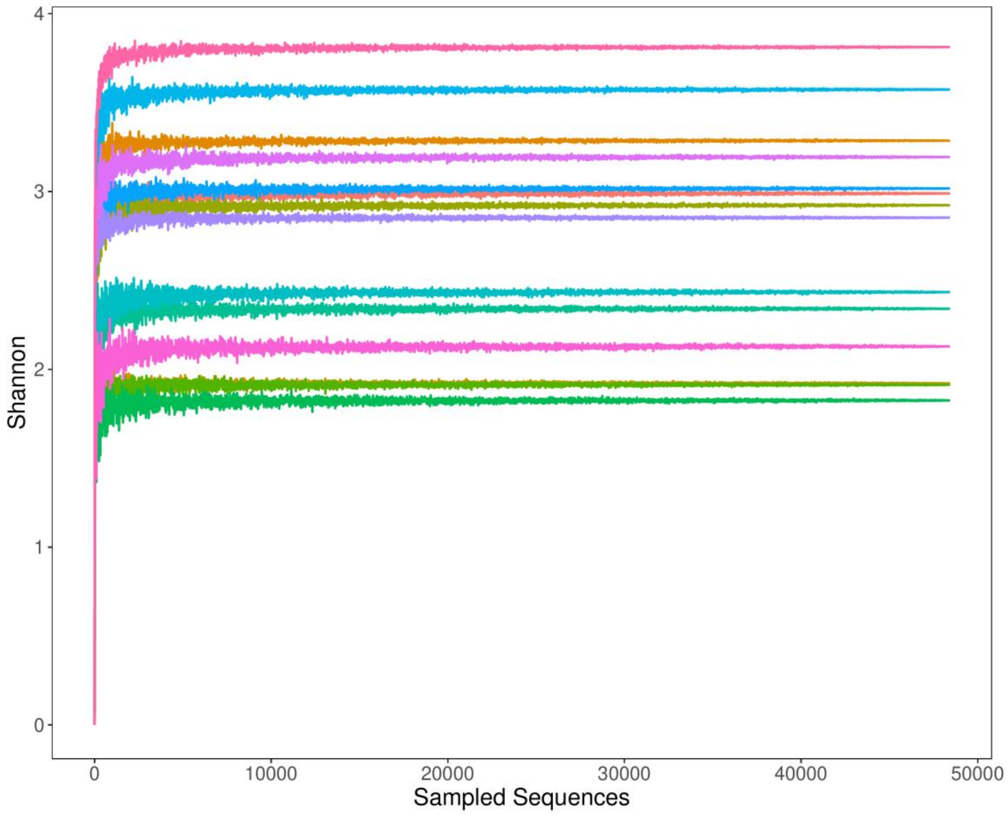

Supplementary Fig. 1: Shannon-Wiener index curve 14 samples from lung cancer patients and healthy controls.

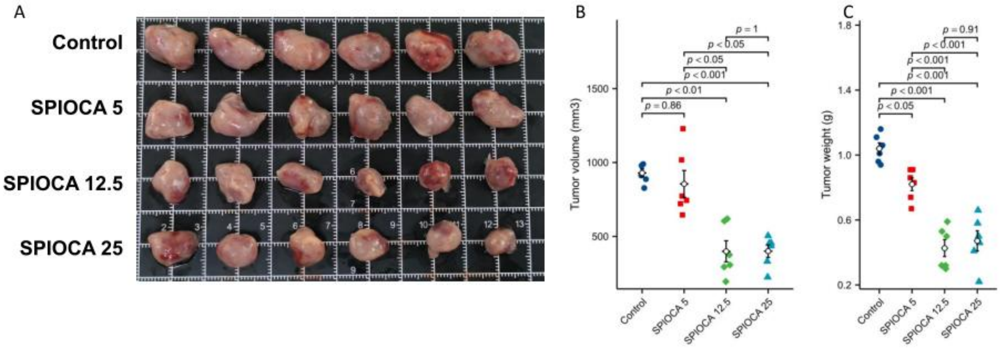

Supplementary Fig. 2: Inhibition of lung cancer by SPIOCA.

A. Tumor images from each group harvested from mice post-euthanasia.

B. Tumor volume comparison across groups after euthanasia.

C. Tumor weight comparison across groups after euthanasia.

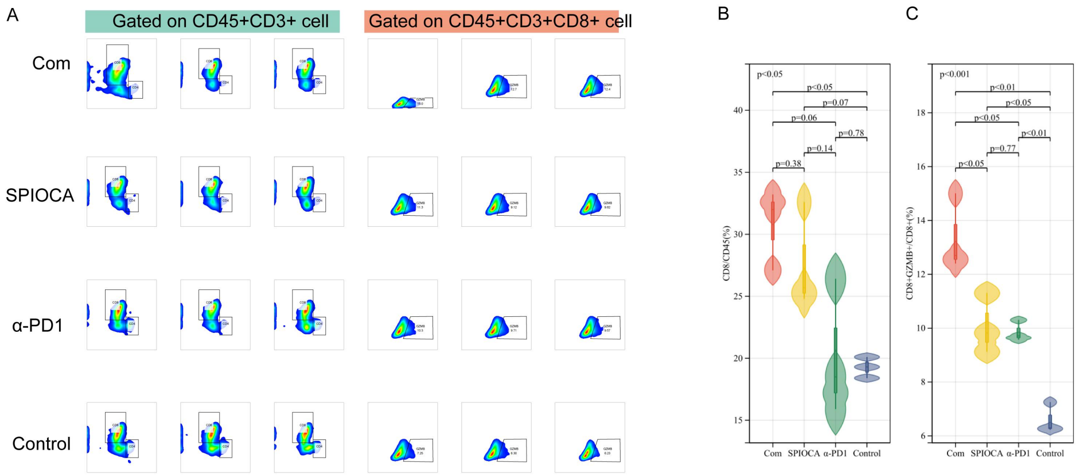

Supplementary Fig. 3: Comparison of T cells in tumors across different groups.

A.Flow cytometric analysis of $CD4^+$ T cells, $CD8^+$ T cells, and $CD8^+GZMB^+$ T cells in tumors from Com-, SPIOCA-, α-PD1-, and Control-treated mice.

B. Quantitative analysis of CD3+CD8+T cells using one-way ANOVA.

C. Quantitative analysis of CD8+GZMB+ T cells using one-way ANOVA.

Supplementary Data 2

Methods

1. Patient recruitment and specimens

Between January and June 2024, 14 adults were enrolled at Shanghai Pulmonary Hospital for fecal sample collection, including 7 lung cancer patients and 7 healthy controls. The exclusion criteria included: age <18 or >75 years; use of antibiotics, probiotics, prebiotics, or other medications that could significantly alter the gut microbiota within the past 4–6 weeks; use of antacids, laxatives, or anti-diarrheal medications; and a history of gastrointestinal surgery or procedures within the past 6 months. Ethical approval for this study was granted by the Research Ethics Committee of Shanghai Pulmonary Hospital (reference number K22-248Z).

2. Animal models

The animals used in this study were 6- to 8-week-old male C57BL/6J mice (SPF level) sourced from Shanghai SLAC Laboratory Animal Co., Ltd. All mice were fed a standard diet and maintained on a 12-hour light/dark cycle at room temperature (22 ± 2°C) with controlled humidity (~45%).

To develop a murine model of gut microbiota disruption, the Abx group was provided with drinking water containing a broad-spectrum antibiotic cocktail. The cocktail consisted of 0.25 g ampicillin, 0.25 g colistin, 1.25 g neomycin, and 0.0625 g vancomycin, dissolved in 250 mL of distilled water. Subcutaneous tumors were established using the murine lung cancer cell line LLC (10^7cells/mL) after 14 days of antibiotic pretreatment. Tumors were induced by injecting 100 μL of the cell suspension into the right dorsal flank of each mouse. All procedures were performed under anesthesia induced by intraperitoneal injection of 12.5% tribromoethanol at a dose of 0.25 mL per 10 g body weight. When the tumor volume reached approximately 20–30 mm^3, treatment was initiated according to the assigned groups. Mice in the anti-PD-1 treatment group received 200 μg of anti-mouse PD-1 antibody (Selleck, A2122, China) via intraperitoneal injection every 3 days, for a total of 4 times in total. In the SPIOCA-treated groups, mice were administered SPIOCA at doses of 5 mg/kg, 12.5 mg/kg, or 25 mg/kg via daily oral gavage throughout the experiment. Tumor volume was calculated using the following formula: volume = (length × $width^2$)/2.

The experimental duration was 3 to 6 weeks, and samples of tumors, feces, blood, and intestinal tissues, as well as organs including the heart, liver, spleen, lungs, and kidneys, were collected.

3. Synthesis of SPIOCA

A total of 300 mg of CMC was dissolved in 40 mL of ultrapure water, and 60 mg/30 mg, 150 mg/75 mg, or 300 mg/150 mg of hexahydrate ferric chloride and tetrahydrate ferrous chloride were dissolved in another 5 mL of ultrapure water. The two solutions were mixed in a three-necked flask and stirred under nitrogen flow (200 rpm) for 30 minutes. The water bath was heated to 60℃, and 5 mL of 14% ammonia

solution was added using a microsyringe (1 mL/min) with vigorous stirring (800 rpm). The temperature of the water bath was then raised to 80℃ and maintained for 30 minutes before stopping the reaction. The resulting SPIOCA was obtained after workup by dialysis and filtration.

4. Cell culture and reagents

LLC cells were obtained from ATCC (Cat. TIB-49) and cultured in Dulbecco's modified Eagle's medium (DMEM) (Invitrogen, Carlsbad, CA) containing 10% fetal bovine serum (FBS) (Sigma-Aldrich, St Louis, MO) and 1% penicillin/streptomycin. Cells were incubated at 37 °C in a humidified atmosphere with 5% CO_2.

The primary antibodies used for Western blot analysis included anti-claudin-1 (A2196), anti-CLDN2 (A14085), anti-CLDN3 (A2946), anti-CLDN7 (A2035), anti-TJP2 (A0594), anti-GAPDH (A19056), and anti-β-Actin, all of which were sourced from ABclonal Biotechnology Co., Ltd. Antibodies against E-Cadherin (#3195) and Occludin (#91131) were obtained from Cell Signaling Technology (Beverly, MA, USA), and anti-ZO-1 (ab221547) was purchased from Abcam (Cambridge, UK). ZO-1 (Servicebio, GB111402,1:500), claudin-1 (Servicebio, GB11032,1:500), Occludin (Abcam, ab53032,1:500), CD8 (ABclonal, A16039, 1:200), DAPI (Servicebio, G1407-25ML) used for immunofluorescence.

5. Immunofluorescence staining

Immunofluorescence (IF) staining was performed using methods similar to those previously described and validated [1]. In summary, formalin-fixed paraffin-embedded tissues were sectioned at 4 μm. Sections were deparaffinized with xylene and rehydrated through an ethanol gradient. Antigen retrieval was achieved using AR6 buffer (Akoya Biosciences) in a microwave. Endogenous peroxidase activity was blocked by treating sections with 3% H_2O_2 for 10 minutes. Multiplex immunohistochemistry was performed in cycles, each involving blocking with 1% BSA, followed by primary and secondary antibody incubations. Secondary antibodies were horseradish peroxidase-conjugated (Akoya Biosciences). Slides were then incubated with Opal fluorophores (1:100) in 1x Plus Diluent (Akoya Biosciences). After signal amplification and fluorophore binding, antibodies were removed by antigen retrieval, and the next cycle began. Primary antibodies were used in the order of anti-CD8 (ABclonal, A16039, 1:200), anti-ZO-1 (Servicebio, GB111402,1:500), anti-claudin-1 (Servicebio, GB11032,1:500), and anti-Occludin (Abcam, ab53032,1:500). Slides were counterstained with spectral DAPI (Servicebio, G1407-25ML) and mounted with an anti-fade medium (ab104135, Abcam).

6. Transmission electron microscopy

Upon euthanasia, intestines were collected from mice in the control, Abx+SPIOCA, and Abx groups. Transverse sections (2 mm thick) were promptly excised from the colon. The sections were washed thrice in Sorensen's buffer with 0.1% ruthenium red

(RR), then post-fixed for 1 hour in 1% osmium tetroxide in the same buffer with RR. After another wash in Sorensen's buffer containing RR, the tissues were dehydrated through an ethanol series, treated with propylene oxide, and embedded in Epon resin. Semithin sections were stained with toluidine blue for histological identification. Ultrathin sections (70 nm) were cut from areas of interest, placed on copper grids, and stained with uranyl acetate and lead citrate. Tissue ultrastructure was examined using a Philips CM100 transmission electron microscope at 100 kV.

7. Western blot

Western blotting was conducted as previously reported. Protein extracts were obtained from tissues using RIPA buffer containing protease and phosphatase inhibitors. Protein concentrations were measured using a BCA Protein Assay Kit (Beyotime, P0012, China). Equal quantities of protein (30 μg) were resolved on 10% SDS-PAGE gels and blotted onto PVDF membranes. The membranes were blocked using 5% nonfat milk for 1.5 hours at room temperature, then probed with primary antibodies overnight at 4 ° C. Subsequently, the membranes were incubated with HRP-conjugated secondary antibodies for 1.5 hours at room temperature. Specific protein bands were visualized using an ECL hypersensitive luminescence reagent (RM00021P, ABclonal, China).

8. Multicolor flow cytometry

Tumors were removed from mice, cut into small pieces, and digested in DMEM with 10% fetal bovine serum, DNase I (0.1 mg/ml), and Collagenase IV (0.5 mg/ml) at 37°C for 30 minutes with gentle shaking. The digested tissues were filtered through 75-μm strainers, and single-cell suspensions were collected by centrifugation at 250 × g for 5 minutes. The cells were washed twice with protein-free PBS. Cells were incubated with Mouse FcR Blocking Reagent (S0B0599-100T) for 30 minutes at 4°C. For viability staining, Zombie NIR™ dye was diluted 1:100-1000 in PBS, and 1-10 × 10^6 cells were incubated in 100 μL of this solution at room temperature in the dark for 15-30 minutes, then washed with BioLegend's Cell Staining Buffer (Cat. No. 420201). For surface staining, cells were incubated with surface antigen antibodies in blocking buffer on ice for 30 minutes. For intracellular cytokine staining, cells were fixed and permeabilized using the Transcription Factor Buffer Set (562574, BD Pharmingen) as per the manufacturer's instructions, then stained with specific antibodies. The specific antibodies used include CD45 (BD Pharmingen, PerCP-Cy5.5, 550994), CD3 (BD Pharmingen, FITC, 553061), CD4 (BD Pharmingen, BV605, 563151), CD8 (BD Pharmingen, PE-Cy7, 552877), GZMB (Biolegend,

Brilliant Violet 421, 396413), CD11c (BD Pharmingen, PE, 557401), and I-A/I-E (Biolegend, Brilliant Violet 650™, 107641).

9. Microbial sequencing

To extract microbial DNA from fecal samples, the E.Z.N.A.® Soil DNA Kit (Omega Bio-tek) was utilized, following the manufacturer's protocol. DNA quantity and purity were measured with a NanoDrop 2000 UV-vis spectrophotometer (Thermo Scientific), and DNA integrity was checked by 1% agarose gel electrophoresis. The V3-V4 regions of the bacterial 16S rRNA gene were amplified via PCR using primers 338F and 806R on a GeneAmp 9700 system (ABI). The PCR protocol included an initial denaturation at 95°C for 3 minutes, followed by 27 cycles of denaturation at 95°C for 30 seconds, annealing at 55°C for 30 seconds, and extension at 72°C for 45 seconds, with a final elongation at 72°C for 10 minutes and hold at 4°C. Each 20 μL reaction mixture contained 4 μL of 5× TransStart FastPfu buffer, 2 μL of 2.5 mM dNTPs, 0.8 μL of each primer (5 μM), 0.4 μL of TransStart FastPfu DNA Polymerase, and 10 ng of template DNA, and PCR was performed in triplicate. The PCR products were purified from a 2% agarose gel using the AxyPrep DNA Gel Extraction Kit (Axygen Biosciences), and their quantity was assessed with the Qubit 4 fluorometer (Thermo Fisher). The purified amplicons were pooled in equimolar amounts and sequenced on the Illumina MiSeq PE300 platform by Genefund Biotech (Shanghai, China) according to standard procedures.

10. Non-targeted metabolomics analysis

GC-MS analysis

Metabolomic profiling was conducted using an Agilent 7890B GC system paired with a 5977A MSD. Derivatized samples were analyzed on a DB-5MS column (30 m × 0.25 mm × 0.25 μm) with helium as the carrier gas at a flow rate of 1 mL/min. The injector was maintained at 260°C, and the injection volume was 1 μL in splitless mode. The GC oven temperature program started at 60°C for 0.5 min, then increased to 125°C at 8°C/min, to 210°C at 5°C/min, to 270°C at 10°C/min, to 305°C at 20°C/min, and held at 305°C for 5 min. The MS quadrupole and ion source were set to 150°C and 230°C, respectively, with an electron impact energy of 70 eV. Mass spectra

were collected in full-scan mode (m/z 50–500) with a solvent delay of 5 min.

LC-MS/MS analysis

Shanghai Luming Biological Technology Co., Ltd. carried out metabolomic analysis using an ACQUITY UPLC I-Class Plus system (Waters Corporation) linked to a Q-Exactive mass spectrometer (Thermo Fisher Scientific). The analysis was executed in both positive and negative ESI modes on an ACQUITY UPLC HSS T3 column (1.8 μm, 2.1 × 100 mm). The gradient elution system utilized water with 0.1% formic acid and acetonitrile with 0.1% formic acid. The gradient program was as follows: 0.01 min, 5% B; 2 min, 5% B; 4 min, 30% B; 8 min, 50% B; 10 min, 80% B; 14 min, 100% B; 15 min, 100% B; 15.1 min, 5% B; 16 min, 5% B. The flow rate was set at 0.35 mL/min, and the column temperature was maintained at 45 °C. Samples were stored at 10 °C, with an injection volume of 2 μL. The mass spectrometer operated within a mass range of m/z 100−1000, with a resolution of 70,000 for full MS scans and 17,500 for HCD MS/MS scans. The collision energy was adjusted to 10, 20, and 40 eV.

1. Parra ER, Uraoka N, Jiang M, et al: Validation of multiplex immunofluorescence panels using multispectral microscopy for immune-profiling of formalin-fixed and paraffin-embedded human tumor tissues. Sci Rep 7:13380, 2017
